# Supplementary material for: Origin of acetylcholine antagonism in ELIC, a bacterial pentameric ligand-gated ion channel
Source: Commun Biol. 2022 Nov 18;5:1264. doi: 10.1038/s42003-022-04227-6 (PMC9674596; doi:10.1038/s42003-022-04227-6)
Supplement: Supplementary file 2 — Supplementary Information [file 42003_2022_4227_MOESM2_ESM.pdf]

communications
biology

SUPPLEMENTARY INFORMATION

Origin of acetylcholine antagonism in ELIC, a bacterial pentameric ligand-gated ion channel

Mykhaylo Slobodyanyuk[1,2,4], Jesús A. Banda-Vázquez[1,2,4], Mackenzie J. Thompson[3], Rebecca A. Dean[1,2], John E. Baenziger[3], Roberto A. Chica[1,2✉], and Corrie J. B. daCosta[1,2✉]

[1]Department of Chemistry and Biomolecular Sciences, University of Ottawa, Ottawa, Ontario Canada. [2]Centre for Chemical and Synthetic Biology, University of Ottawa, Ottawa, Ontario Canada. [3]Department of Biochemistry, Microbiology, and Immunology, University of Ottawa, Ottawa, Ontario, Canada.

[4]These authors contributed equally to this work.

✉Correspondence and requests for materials should be addressed to R.A.C. or C.J.B.d.C.

Email: rchica@uottawa.ca, cdacosta@uottawa.ca

Author Contributions: M.S. and M.J.T. acquired and analyzed all electrophysiology data. J.A.B.V. and R.A.D. performed statistical coupling analysis, J.A.B.V. identified sites for mutagenesis. J.A.B.V., M.J.T. and M.S. prepared the DNA constructs. M.J.T. and J.E.B. provided the *Xenopus* oocytes, two-electrode voltage-clamp apparatus, and assisted in technical aspects of the electrophysiology experiments. C.J.B.d.C, R.A.C, J.A.B.V, M.J.T., and M.S. designed the experiments and interpreted the data. C.J.B.d.C, J.A.B.V and M.S. wrote the manuscript. C.J.B.d.C and R.A.C supervised the project. All authors were involved in manuscript editing.

This PDF file includes:

Supplementary Figures 1 to 10
Supplementary Tables 1 to 3

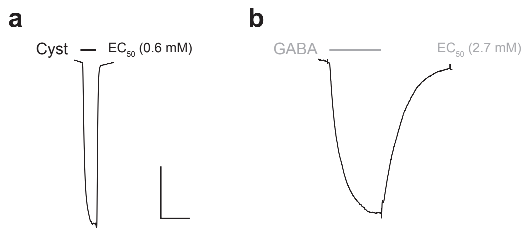

Supplementary Figure 1 | GABA produces slow whole-cell activation/deactivation currents in wild-type ELIC. Representative whole-cell traces are shown for ELIC activated by **(a)** cysteamine (Cyst) and **(b)** GABA at their respective EC_{50} concentrations (in brackets). Duration of cysteamine (black bar) and GABA (gray bar) applications are indicated above each peak. The x- and y-axis of the depicted scale bar in “a” applies to both panels and corresponds to 1 min and 0.5 µA, respectively.

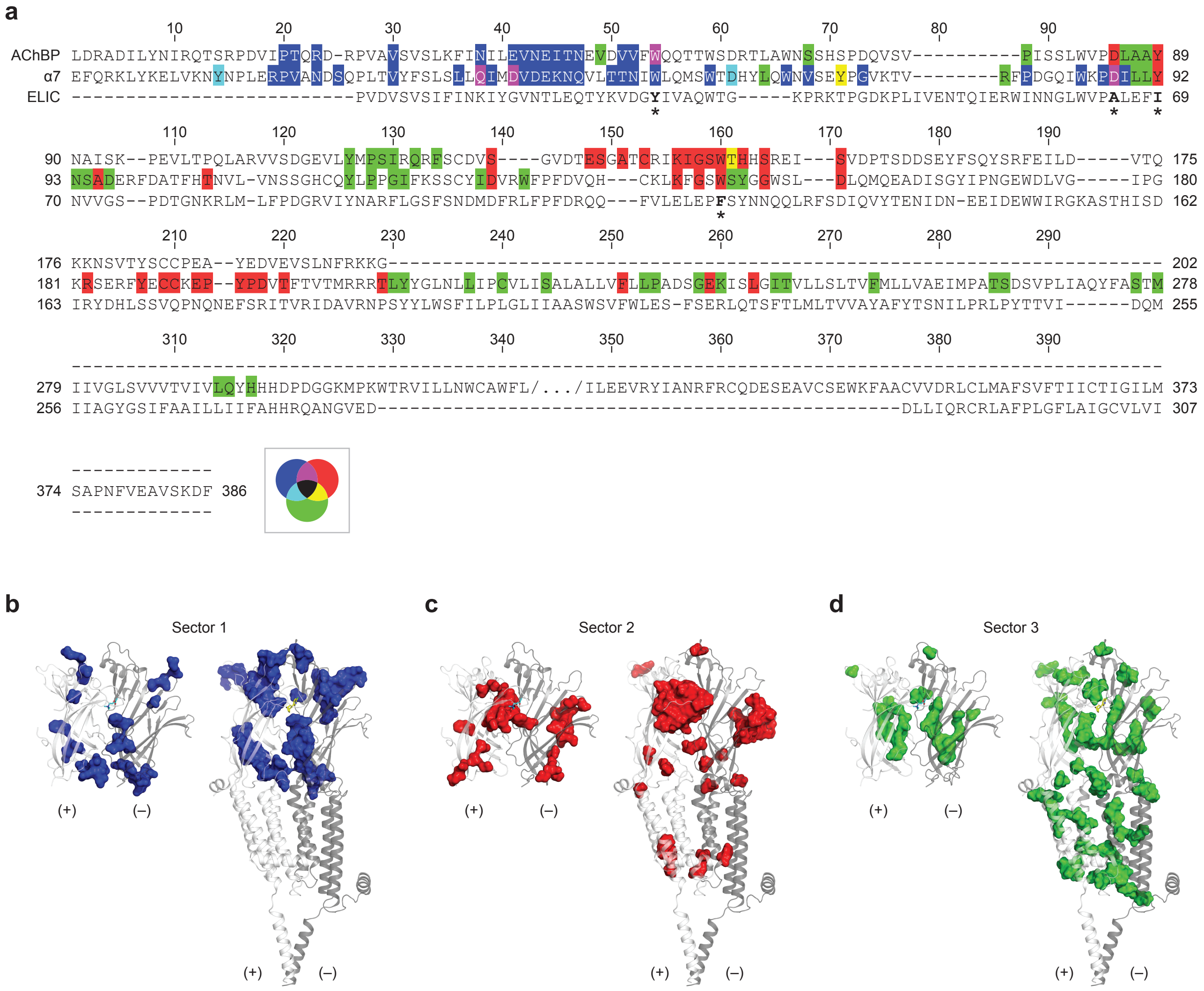

Supplementary Figure 2 | Results of statistical coupling analysis. (a) Sequence alignment between the *Lymnaea stagnalis* acetylcholine binding protein (AChBP), the human α7 acetylcholine receptor (α7), and ELIC. Residues comprising sector 1 (blue), sector 2 (red), and sector 3 (green) are highlighted, while residues found in more than one sector are depicted in cyan, magenta, and yellow (see Venn diagram; boxed inset at the end of the alignment). The four ELIC positions that were mutated in the present work are shown in bold, and indicated with an asterisk (*). Note that the region comprising the cytoplasmic domain of α7, which is missing in the cryo-EM structure, falls between the slashes and is denoted with dots (approximately position 340 in the alignment). **(b-d)** Sector residues identified by statistical coupling analysis are shown as surfaces mapped onto the structure of the AChBP from *Lymnaea stagnalis* (left; PDB ID: 3WIP)[11] and the human α7 acetylcholine receptor (right; PDB ID: 7KOX). Each sector is depicted on both the principal (+) and complementary (–) subunits encompassing a single agonist-binding site.

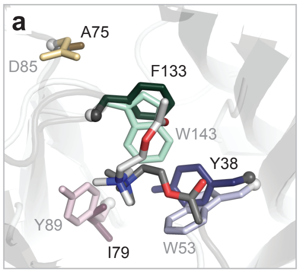

Supplementary Figure 3 | Comparing the binding site between acetylcholine binding protein (AChBP) from *Lymnaea stagnalis* and ELIC. (a) Crystal structure (PDB ID: 3RQW)[10] of ELIC and the *Lymnaea stagnalis* AChBP (PDB ID: 3WIP)[11] both in complex with acetylcholine. The backbone (cartoon) and bound acetylcholine (sticks) are coloured in dark gray in ELIC and light gray in AChBP. ELIC Y38 (dark purple), A75 (dark orange), I79 (dark magenta) and F133 (dark green) Cα atoms were aligned with the Cα atoms of W53 (light purple), D85 (light orange), Y89 (light magenta) and W143 (light green) in the AChBP structure. RMSD between the two sets of Cα atoms is 0.46 Å.

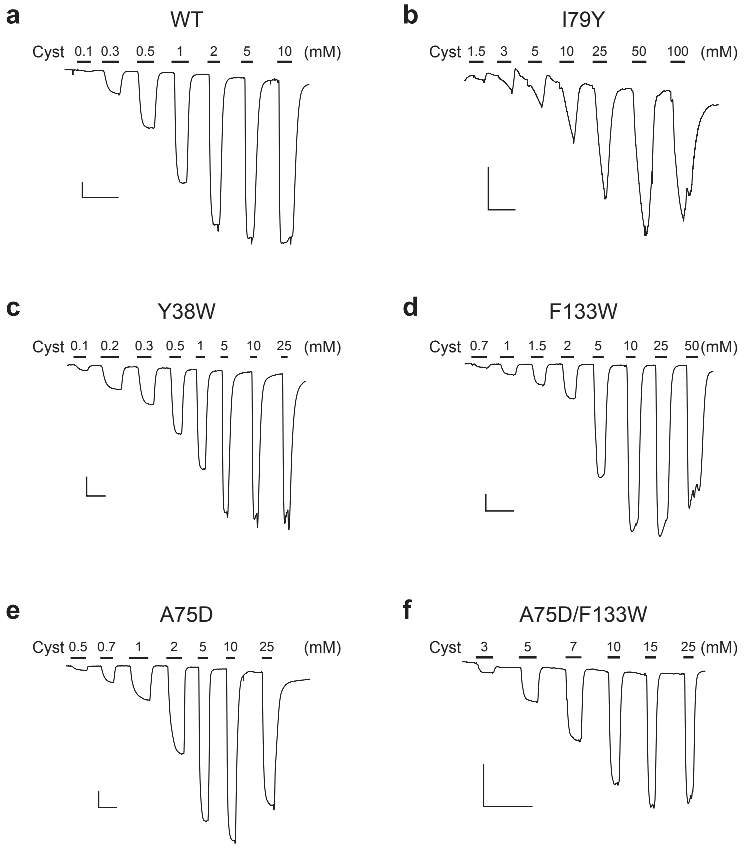

Supplementary Figure 4 | Cysteamine responses of wild-type and ELIC mutants. Representative whole-cell traces for **(a)** wild-type, **(b)** I79Y, **(c)** Y38W, **(d)** F133W, **(e)** A75D, and **(f)** A75D/F133W variants of ELIC are provided (minimum of 6 independent oocyte replicates for each). Duration of cysteamine application (black bars) and concentration (in mM) are indicated above each trace. In each panel, the x- and y-axis of the depicted scale bar corresponds to 1 min and 0.5 μA, respectively.

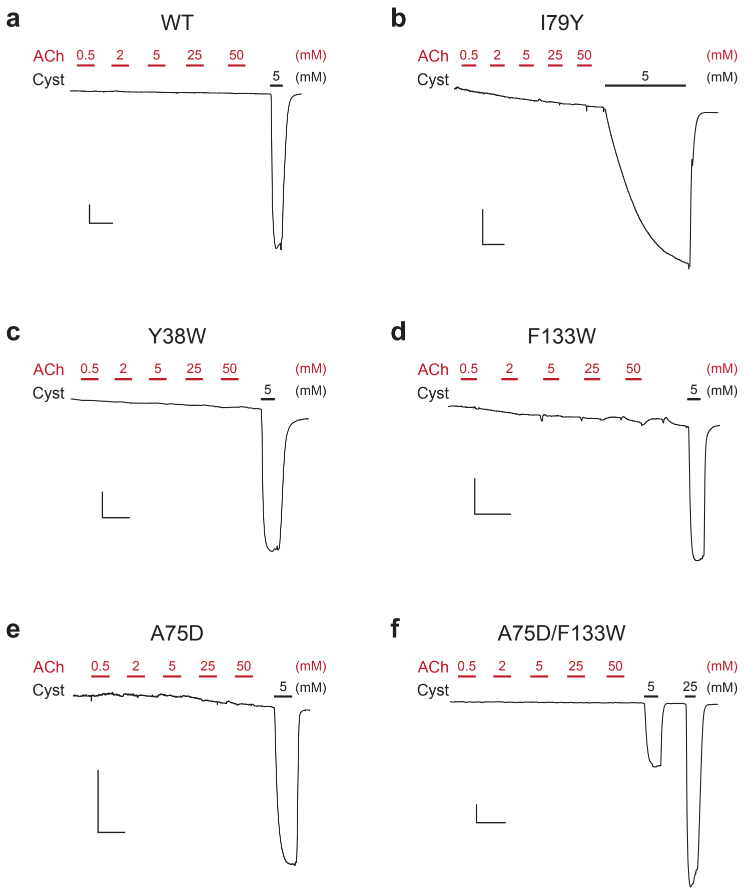

Supplementary Figure 5 | Response of wild-type and various ELIC mutants to acetylcholine. Representative whole-cell traces for **(a)** wild-type, **(b)** I79Y, **(c)** Y38W, **(d)** F133W, **(e)** A75D, and **(f)** A75D/F133W variants of ELIC are provided (minimum of 3 independent oocyte replicates for each). Acetylcholine (ACh) and cysteamine (Cyst) concentrations (in mM) are indicated above each peak with the duration of each application shown as red (ACh) or black (Cyst) bars. In each panel, the x- and y-axis of the depicted scale bar corresponds to 1 min and 0.5 μA, respectively.

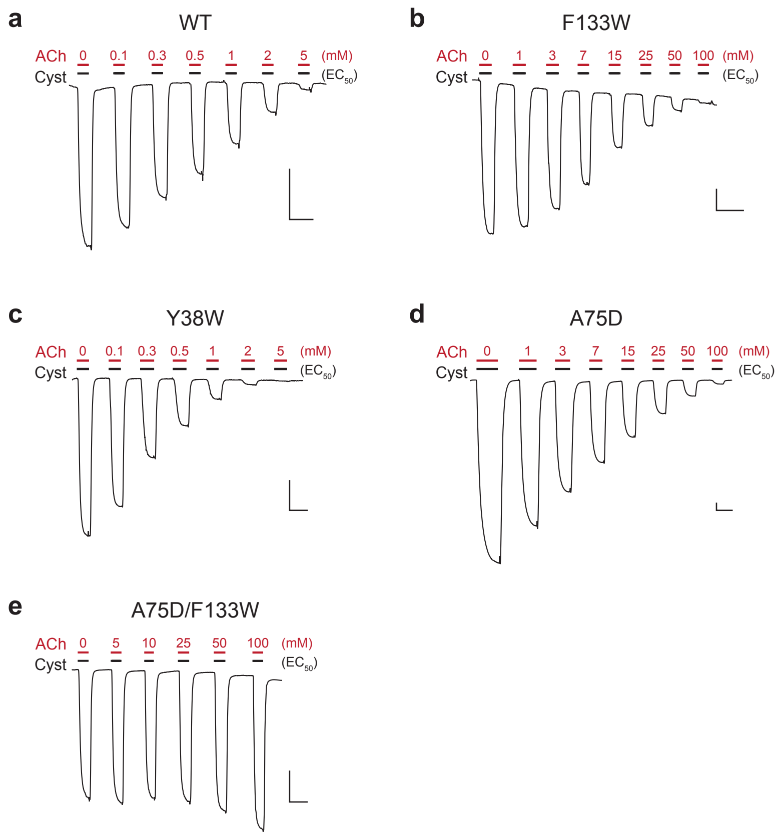

Supplementary Figure 6 | Acetylcholine inhibition of cysteamine-activated currents for various mutants and wild-type ELIC. Representative whole-cell traces for **(a)** wild-type, **(b)** F133W, **(c)** Y38W, **(d)** A75D, and **(e)** A75D/F133W variants of ELIC are provided (minimum of 5 independent oocyte replicates for each). Duration of acetylcholine (ACh; red bars) and cysteamine (Cyst; black bars) applications are indicated above each peak, as is the concentration (in mM) of acetylcholine added. The cysteamine concentration was fixed at its EC_{50} value (Table 1) for each mutant. In each panel, the x- and y-axis of the depicted scale bar corresponds to 1 min and 0.5 μA, respectively.

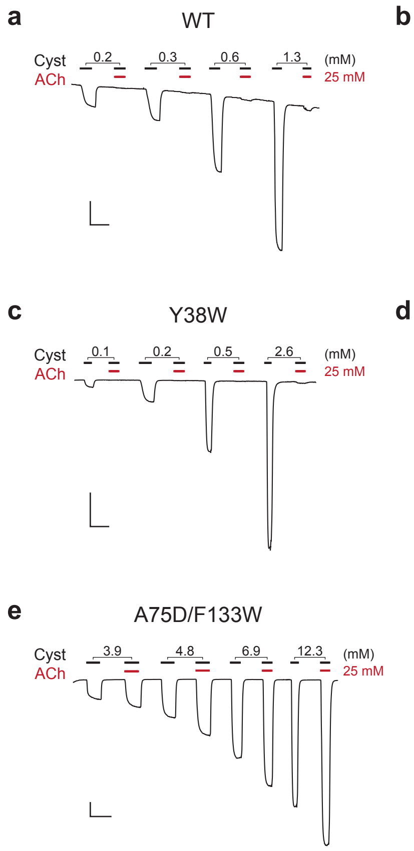

Supplementary Figure 7 | Acetylcholine inhibition of wild-type and mutant ELIC variants activated by increasing cysteamine concentrations. The concentration of cysteamine (in mM) corresponding to EC_{10}, EC_{20}, EC_{50} and EC_{90} for **(a)** wild-type, **(b)** F133W, **(c)** Y38W, **(d)** A75D, and **(e)** A75D/F133W variants of ELIC are indicated (Cyst; black bars), as is the application of 25 mM acetylcholine (ACh; red bars). Representative whole-cell traces for each variant are provided (minimum of 4 independent oocyte replicates for each). In each panel, the x- and y-axis of the scale bar corresponds to 1 min and 0.5 μA, respectively.

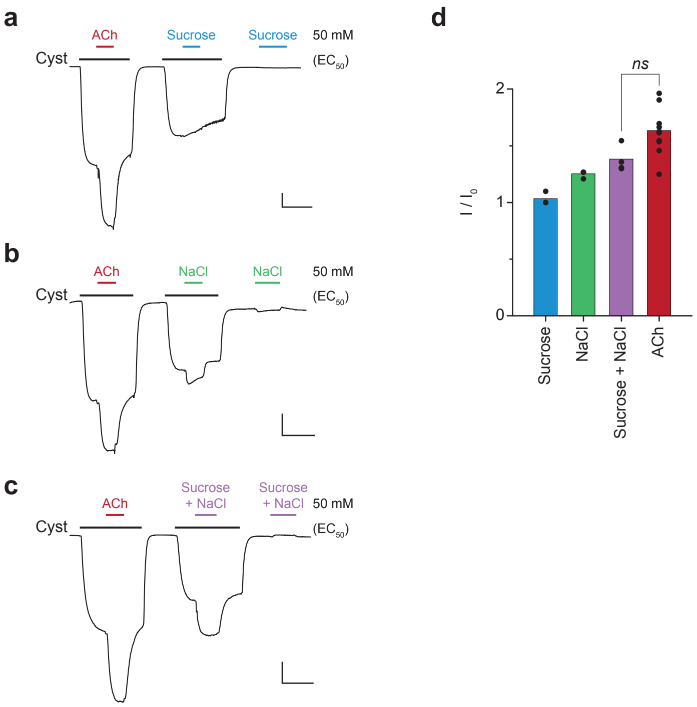

Supplementary Figure 8 | Apparent potentiation of the A75D/F133W double mutant ELIC by acetylcholine, sucrose, and excess sodium chloride (NaCl). (a-c) Representative traces for each condition are provided. The first peak depicts a 2 min cysteamine pulse at its EC_{50} (6.9 mM) during which a pulse of 50 mM acetylcholine (ACh) was co-applied. The second peak depicts another 2 min cysteamine pulse at its EC_{50} during which **(a)** 50 mM sucrose, **(b)** an additional 50 mM NaCl, or **(c)** 50 mM sucrose with an additional 50 mM NaCl were co-applied. After the second peak **(a)** 50 mM sucrose, **(b)** 50 mM NaCl, or **(c)** 50 mM sucrose with an additional 50 mM NaCl were applied by themselves for 40 s producing a negligible response. In each panel, the x- and y-axis of the depicted scale bar corresponds to 1 min and 0.5 μA, respectively. **(d)** Bar graph depicting the normalized mean response (I/I_0) on the y-axis and the chemicals co-applied with cysteamine on the x-axis. The normalized mean response was calculated by dividing the magnitude of the peak current elicited by cysteamine with 50 mM chemical (sucrose, NaCl, sucrose + NaCl, or ACh) versus cysteamine alone before chemical application. Individual data points are shown (n = 3, 3, 4, and 10 for the sucrose, NaCl, sucrose + NaCl, and ACh conditions, respectively. For the sucrose and NaCl conditions two of the data points overlap perfectly and are thus indistinguishable). Note that the difference in mean potentiation by ACh vs. sucrose + NaCl is not statistically significant based on a one-way ANOVA with a Dunnett post hoc test.

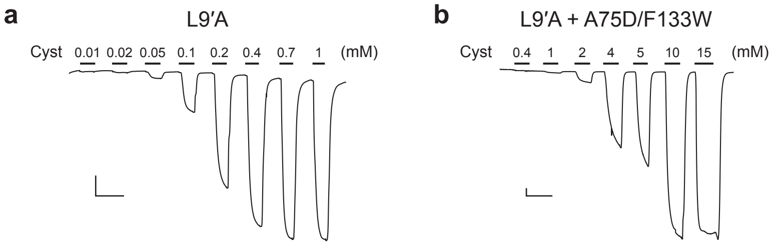

Supplementary Figure 9 | Cysteamine responses of the L9′A mutants. Representative whole-cell traces for **(a)** L9′A and **(b)** L9′A + A75D/F133W are provided (minimum of 6 independent oocyte replicates for each). Duration of cysteamine application (black bars) and concentration (in mM) are indicated above each trace. In each panel, the x- and y-axis of the scale bar corresponds to 1 min and 0.5 μA, respectively.

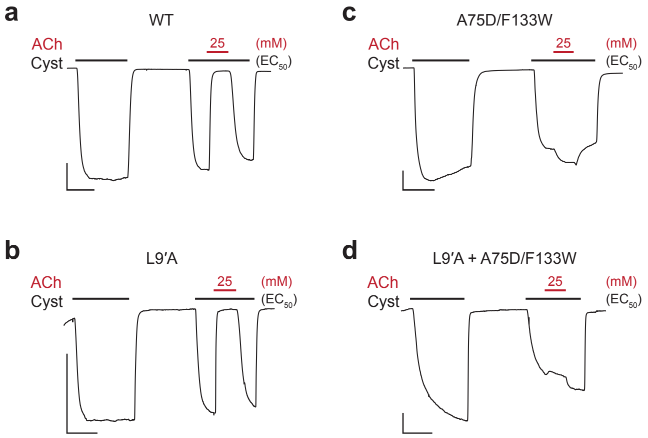

Supplementary Figure 10 | The ELIC A75D/F133W double mutant drastically attenuates ACh inhibition in both wild-type and L9′A backgrounds. Representative whole-cell traces for **(a)** wild-type (WT), **(b)** L9′A, **(c)** A75D/F133W, and **(d)** L9′A + A75D/F133W. In each panel, the first peak shows the response to a 2 min application of cysteamine at its corresponding EC_{50} (black bar). The second peak shows the response to another 2 min application of cysteamine, again at its EC_{50}, but which was interrupted by a 40 s pulse where 25 mM ACh was added (red bar). In each case, the x- and y-axis of the depicted scale bar corresponds to 1 min and 0.5 μA, respectively.

Supplementary Table 1: Summary of statistics for comparisons between log(EC_{50}) of activation.

Dunnett's Multiple Comparison Test	Mean Difference (log10 M)	q-score	p< 0.001?	99.9% CI of mean difference (log10 M)
WT (11) vs. Y38W (8)	0.05	1.21	No	-0.12 to 0.22
WT (11) vs. A75D (9)	-0.44	11.04	Yes	-0.60 to -0.28
WT (11) vs. I79Y (6)	-1.39	31.31	Yes	-1.57 to -1.21
WT (11) vs. F133W (8)	-0.87	21.17	Yes	-1.04 to -0.70
WT (11) vs. A75D/F133W (7)	-1.09	25.57	Yes	-1.26 to -0.91
WT (11) vs. L240A (L9′A) (8)	0.58	14.08	Yes	0.41 to 0.75
WT (11) vs. L240A (L9′A) + A75D/F133W (6)	-0.89	20.09	Yes	-1.07 to -0.71
WT Cysteamine (11) vs. WT GABA (6)	-0.68	15.37	Yes	-0.86 to -0.50

Note: "WT" indicates wild-type ELIC. Unless otherwise indicated all EC_{50} data were acquired using cysteamine as the agonist. Sample size is indicated in parentheses.

Supplementary Table 2: Summary of statistics for comparisons between log(IC_{50}) of acetylcholine inhibition.

Dunnett's Multiple Comparison Test	Mean Difference (log10 M)	q-score	p< 0.001?	99.9% CI of mean difference (log10 M)
WT (8) vs. Y38W (8)	0.44	8.66	Yes	0.23 to 0.65
WT (8) vs. A75D (6)	-0.83	15.26	Yes	-1.04 to -0.61
WT (8) vs. F133W (8)	-0.91	18.00	Yes	-1.11 to -0.70
WT Cysteamine (8) vs. WT GABA (5)	-0.03	0.49	No	-0.27 to 0.21

Note: "WT" indicates wild-type ELIC. Unless otherwise indicated all IC_{50} data were acquired using cysteamine as the agonist. Sample size is indicated in parentheses.

Supplementary Table 3: Summary of statistics for comparisons between apparent potentiation of the A75D/F133W mutant.

Dunnett's Multiple Comparison Test	Mean Difference	q-score	p < 0.001?	99.9% CI of mean difference
Acetylcholine (10) vs. Sucrose (3)	0.60	5.41	Yes	0.31 to 0.88
Acetylcholine (10) vs. NaCl (3)	0.38	3.44	Yes	0.09 to 0.67
Acetylcholine (10) vs. Sucrose+NaCl (4)	0.25	2.52	No	-0.01 to 0.51

Note: Sample size is indicated in parentheses.
