# Supplementary material for: Origin of acetylcholine antagonism in ELIC, a bacterial pentameric ligand-gated ion channel
Source: Commun Biol. 2022 Nov 18;5:1264. doi: 10.1038/s42003-022-04227-6 (PMC9674596; doi:10.1038/s42003-022-04227-6)
Supplement: Supplementary file 3 — Reporting Summary [file 42003_2022_4227_MOESM3_ESM.pdf]

nature portfolio

Corresponding author(s): Corrie daCosta & Roberto Chica

Last updated by author(s): Oct 25, 2022

Reporting Summary

Nature Portfolio wishes to improve the reproducibility of the work that we publish. This form provides structure for consistency and transparency in reporting. For further information on Nature Portfolio policies, see our Editorial Policies and the Editorial Policy Checklist.

Statistics

For all statistical analyses, confirm that the following items are present in the figure legend, table legend, main text, or Methods section.

n/a	Confirmed	
☐	☒	The exact sample size (*n*) for each experimental group/condition, given as a discrete number and unit of measurement
☐	☒	A statement on whether measurements were taken from distinct samples or whether the same sample was measured repeatedly
☐	☒	The statistical test(s) used AND whether they are one- or two-sided<br>*Only common tests should be described solely by name; describe more complex techniques in the Methods section.*
☒	☐	A description of all covariates tested
☒	☐	A description of any assumptions or corrections, such as tests of normality and adjustment for multiple comparisons
☐	☒	A full description of the statistical parameters including central tendency (e.g. means) or other basic estimates (e.g. regression coefficient) AND variation (e.g. standard deviation) or associated estimates of uncertainty (e.g. confidence intervals)
☒	☐	For null hypothesis testing, the test statistic (e.g. *F*, *t*, *r*) with confidence intervals, effect sizes, degrees of freedom and *P* value noted<br>*Give P values as exact values whenever suitable.*
☒	☐	For Bayesian analysis, information on the choice of priors and Markov chain Monte Carlo settings
☒	☐	For hierarchical and complex designs, identification of the appropriate level for tests and full reporting of outcomes
☒	☐	Estimates of effect sizes (e.g. Cohen's *d*, Pearson's *r*), indicating how they were calculated

Our web collection on statistics for biologists contains articles on many of the points above.

Software and code

Policy information about availability of computer code

Data collection

1. Statistical Coupling Analysis Toolbox.
- Statistical Coupling Analysis was performed using the SCA v5.0 Toolbox from the Rama Ranganathan Group, which is available as stated in McLaughlin et al., 2012 (https://rdcu.be/cGnEq) in the Online Methods section. Based on the tutorial scripts from the SCA v5.0 toolbox, a custom script was made for our particular case, and this is provided along with our work. In order to use the toolbox and run our script, we used MATLAB (R2019a) with the additional Bioinformatics and Statistics and Machine Learning toolboxes; reproducing the results with more recent MATLAB versions like R2021a and R2021b.

2. Electrophysiology Experiments.
- Electrophysiology data was acquired with Labscribe 2.

Data analysis

The structural superposition between AChBP (PDB 3WIP), human α7 AChR (PDB 7KOX), and ELIC (PDB 3RQW), as well as the derived pairwise alignment and visualization of statistically coupled positions on structure were done in PyMOL 1.8.4.0. We used Rosetta 3.9 for the sequence redesign of PDB 3WIP and 7KOX.
HMMER 3.2.1 was used to generate the Hidden Markov model out of the redesigned sequences, the sequence search and also to generate the multiple sequence alignment. CD-HIT 4.8.1 was used to filter sequence redundancy. Jalview 2.11.1.0 was used for initial alignment visualization.

GraphPad Prism (v.8.0.0) and R (v.4.0.0) were used for creating the electrophysiology traces, dose-response curves and bar graphs. Chemical structure formulas were prepared in ChemDraw (v.20.1.1). Protein structures were visualized in PyMOL (1.8.4.0).
All figures were prepared with Adobe Illustrator CS3 (25.1).

For manuscripts utilizing custom algorithms or software that are central to the research but not yet described in published literature, software must be made available to editors and reviewers. We strongly encourage code deposition in a community repository (e.g. GitHub). See the Nature Portfolio guidelines for submitting code & software for further information.

Data

Policy information about availability of data

All manuscripts must include a data availability statement. This statement should provide the following information, where applicable:

- Accession codes, unique identifiers, or web links for publicly available datasets
- A description of any restrictions on data availability
- For clinical datasets or third party data, please ensure that the statement adheres to our policy

Source data are provided with this paper. (https://figshare.com/articles/dataset/MSA_and_SCA_script_for_commsbio_2022/19127057)

Field-specific reporting

Please select the one below that is the best fit for your research. If you are not sure, read the appropriate sections before making your selection.

☒ Life sciences ☐ Behavioural & social sciences ☐ Ecological, evolutionary & environmental sciences

For a reference copy of the document with all sections, see nature.com/documents/nr-reporting-summary-flat.pdf

Life sciences study design

All studies must disclose on these points even when the disclosure is negative.

Sample size	All electrophysiology experiments were performed with a minimum of 3 oocytes and a maximum of 11.
Data exclusions	All electrophysiology data is included, unless the recording produced unmeasurable currents or the voltage clamp was lost during the experiment. Exclusion criteria was pre-established.
Replication	Experiments were repeated to achieve a minimum of 3 replicates, which produced consistent results. Where appropriate, the results are reported as an average with standard deviation (shown as error bars in figures).
Randomization	Wild-type and mutant oocytes were studied separately, without randomization, to discern the functional impact of these mutations. There are no covariates in the study.
Blinding	Blinding was not used, because the identity of each mutation was crucial to study its impact on channel function.

Reporting for specific materials, systems and methods

We require information from authors about some types of materials, experimental systems and methods used in many studies. Here, indicate whether each material, system or method listed is relevant to your study. If you are not sure if a list item applies to your research, read the appropriate section before selecting a response.

Materials & experimental systems

n/a	Involved in the study	
☒	☐	Antibodies
☐	☒	Eukaryotic cell lines
☒	☐	Palaeontology and archaeology
☒	☐	Animals and other organisms
☒	☐	Human research participants
☒	☐	Clinical data
☒	☐	Dual use research of concern

Methods

n/a	Involved in the study	
☒	☐	ChIP-seq
☒	☐	Flow cytometry
☒	☐	MRI-based neuroimaging

Eukaryotic cell lines

Policy information about cell lines

Cell line source(s)	Xenopus laevis oocytes from an adult female frog were used for all electrophysiology experiments, which were performed in the Baenziger lab. The Baenziger lab does not purchase Xenopus frogs, but obtains the oocytes from the Johnny Liu laboratory. Therefore, no ethical approval for the Baenziger lab was required.
Authentication	Xenopus oocytes were not authenticated.
Mycoplasma contamination	Oocytes were not tested for mycoplasma contamination, and no human cell lines were used in this study.

Commonly misidentified lines<br>(See ICLAC register)	Not applicable.
